# Collectanea Medica, Consisting of Anecdotes, Facts, Extracts, Illustrations, Queries, Suggestions, &c.

**Published:** 1815-02

**Authors:** 


### COLLECTANEA MEDICA,

CONSISTING OF

### ANECDOTES, FACTS, EXTRACTS, ILLUSTRATIONS, QUERIES, SUGGESTIONS, &c.

RELATING TO THE

***History or the Art of Medicine, and the Auxiliary Sciences.***

---

***Memoirs of the celebrated Dr. Harvey, from Notes collected from the Bodleian Library and Ashmolean Museum.***

GUL. HARVEUS, An. ætat. 10, in Schola Cantuar. primis doctrinæ rudimentis imbutus; 14, Col. Gonvil. et Caii Alumnus; 19, peragravit Galliam et Italiam; 23, Patavii Præceptores habuit Eust. Radium, Tho. Minad. H. Fab. ab Aquapend. Consul Angl. 16* fit; 24, Doctor Med. et Chirurg. Reversus Lond. praxin exercuit. et uxorem† duxit; 25, Coll. Med. Socius; 37, Anatom. et Chirurg. Professor; 54, Medicus Regius factus. Scripsit de Motu Sanguinis, et de Gen. Animal. Obiit 30 Jun. MD.CLVII.

---

* Sic. Edit. † Smyth.

Ætat.

Ætat. 80.* (But I well remember that Dr. Alsop, at his funeral sayd, that he was 80 wanting one; and that he was the eldest of 9 brethren.)

He lies buried in a vault at Hempsted in Essex, wch. his brother Eliab Harvey built, he is lapt in lead, and on his brest in great letters DR. WILLIAM HARVEY. I was at his funerall, and helpt to carry him into the vault.

In the library at the Physitians' Colledge, was the following inscription above his statue, (which was in his doctorall robes).

GUL. HARVEUS, Natus A.D. 1578, Apr. 2. Folkston,† in Com. Cantii, Primogenitus Tho. Harvei et Joanæ Halk. Frat. Germani. Tho. Jo. Dan. Eliab. Mich. Mat. Sorores. Sarah, Amey.

Under his white marble statue, on the pedestall, thus,

GULIELMO HARVEO,
VIVO,
Monumentis suis immortalli,
Hoc insuper
Coll. Med. Lond.
Posuit
Qui enim SANGUINIS MOTUM
(ut et ANIMAL ORTUM) dedit
meruit esse
STATOR Perpetuus.

Dr. Harvey added (or was very bountifull in contributing to) a noble building of Roman architecture (of rustique work with Corinthian pillasters) at the Physitians' College aforesaid, viz. a great parlour, a kind of convocation-house for the fellows to meet in belowe; and a library above. On the outside, on the freeze, in letters 3 inches long, is this inscription, SUASU ET CURA FRAN. PRUJEANI, PRÆSIDIS, ET EDMUNDI SMITH, ELECT. INCHOATA ET PERFECTA EST HÆC FABRICA. AN. MIↃCLIII.

All these buildings and remembrances were destroyed by the generall fire.

He was always very contemplative, and the first yt. I heare of yt. was curious in Anatomie in England. He had made dissections of froggs, toades, and a number of other animals, and had curious observations on them, which papers, together with his goods, in his lodgings at White-hall, were plundered at the beginning of the rebellion, he being for the king, and with him at Oxon; but he

---

* Over Dr. Harvey's picture in the great parlour under the library, at the Physitians' College at Amen-corner, (burnt.)

† Borne in the house which is now the post house, a fair stone built house, which he gave to Caius Coll. in Cambridge, with some lands there, in his will. His brother Eliab would have given any money or exchange for it, because 'twas his father's and they all borne there, but the doctor (truly) thought his memory would better be preserved this way, for his brother has left noble seates, and about £3000 per annum at least.

often often sayd, that of all the losses he sustained, no griefe was so crucifying to him as the losse of these papers, wch. for love or money he could never retrieve or obtaine. When K. Ch. I. by reason of the tumults left London, he attended him, and was at the fight of Edge-hill with him; and, during the fight, the Prince and D. of Yorke were committed to his care. He told me, that he withdrew with them under a hedge, and tooke out of his pockett a booke and read; but he had not read very long before a bullet of a great gun grazed on the ground neare him, which made him remove his station; he told me yt. Sir Adrian Scrope was dangerously wounded there, and left for dead amongst the dead men, stript, which happened to be the saving of his life. It was cold clear weather, and a frost that night; which staunched his bleeding, and about midnight, or some hours after his hurt, he awaked, and was faine to drawe a dead body upon him for warmeth sake.

After Oxford was surrendered, which was 24 July, 1646, he came to London, and lived with his brother Eliab, a rich merchant in London, on —— hill, opposite to St. Lawrence, Poultry, where was then a high leaden steeple, (there were but two, viz. this and St. Dunstan's in the East, and at his brother's country house at Roehampton. His brother Eliab bought, about 1654, Cockaine-house, now (1680) the Excise Office, a noble house, where the doctor was wont to contemplate on the leads of the house, and had his severall stations, in regard of the sun, or wind. He did delight to be in the darke, and told me he could then best contemplate.

He had a house heretofore at Combe, in Surrey, a good air and prospect, where he had caves made in the earth, in which in summer time he delighted to meditate. He was pretty well versed in mathematiques, and had made himselfe master of Mr. Oughtred's Clavis Math. in his old age; and I have seen him perusing it, and working problems not long before he dyed, and that book was always in his meditating apartment. His chamber was that room which is now the office of Elias Ashmole, esq. where he dyed, being taken with the dead palsey, which took away his speech; as soon as he was attaqued, he presently sent for his brother and nephews, and gave one a watch, another another thing, &c. as remembrances of him. He dyed worth £20,000, wch. he left to his brother Eliab. In his will, he left his old friend, Mr. Tho. Hobbes, £10, as a token of his love.

He was wont to say, that man was but a great mischievous baboon.

He would say, that we Europeans know not how to order or govern our woemen, and that the Turkes were the only people [who] used them wisely.

He had been physitian to the Lord Ch. Bacon, whom he esteemed much for his witt and style, but would not allow him to be a great philosopher. Said he to me, "He writes philosophy like a Ld. Chancellor," speaking in derision.*

About

* This must relate to Bacon's physiological opinions as exemplified

About 1649, he travelled again into Italy, Dr. George, now Sir George Ent, then accompanying him.

At Oxford, he grew acquainted with Dr. Charles Scarborough, then a young physitian, (since by Ch. II. knighted) in whose conversation he much delighted; and whereas before, he marched up and downe with the army, he took him to him and made him ly in his chamber, and said to him, "Prithee leave off thy gunning, and stay here, I will bring thee into practice." I remember he kept a pretty young wench to wayte on him, wch. I guesse he made use of for warmth's sake, and tooke care of her in his will, as also of his man servant. For 20 years before he dyed, he tooke no manner of care about his worldly concerns, but his brother Eliab, who was a very wise and prudent manager, ordered all not only faithfully, but better than he could have done for himselfe. He was, as all the rest of the brothers, very cholerique, and in his younger days wore a dagger (as the fashion then was, nay I remember my old schoolmaster, Mr. Latimer, at 70, wore a dudgeon, with a knife and bodkin, as also my old grandfather, Lyte, and Alderman Whitson, of Bristowe, wch. I suppose was the common fashion in their young dayes), but this Dr. would be apt to drawe out his dagger upon every slight occasion.

He was not tall, but of the lowest stature, round faced, olivaster (like wainscott) complexion; little eie, round, very black, full of spirit; his haire was black as a raven, but quite white 20 yeares before he dyed.

I first sawe him at Oxford, 1642, after Edgehill fight, but was then too young to be acquainted with so great a doctor. I remember he came severall times to our Coll. (Trin.) to George Bathurst, B. D. who had a hen to hatch egges in his chamber, which they dayly opened to see the progress and way of generation. I had not the honour to be acquainted [with] him till 1651, being my cos. Montague's physitian and friend. I was at that time bound for Italy, (but to my great grief dissuaded by my mother's importunity). He was very communicative and willing to instruct any that were modest and respectfull to him. And in order to my journey, dictated to me what to see, what company to keep, what bookes to read, how to manage my studyes; in short, he bid me go to the fountaine head, and read Aristotle, Cicero, Avicenna, and did call the neoteriques s . . t breeches. He wrote a very bad hand, which with use I could pretty well read. I have heard him say, that after his booke of the Circulation of the Blood came out, he fell mightily in his practice, and 'twas believed by the vulgar,

---

fied in his *Historia Vitæ & Mortis*, the work which produced so much wit in the Tristram Shandy, concerning radical heat and radical moisture. Harvey's mode of inquiry was exactly such as Bacon pointed out in his Nov. Organum. But it must be admitted, that Bacon's only physiological work savours much of precedents, in the Lord Chancellor Style.—Edit.

that that he was crack-brained; and all the physitians were against his opinion, and envyed him; with much adoe at last in about 20 or 30 yeares time, it was received in all the universities in the world, and, as Mr. Hobbes sayes in his book, "De Corpore," he is the only man, perhaps, that ever lived to see his owne doctrine established in his life-time.

He understood Greek and Latin pretty well, but was no critique, and he wrote very bad Latin. The Circuitus Sanguinis was, as I take it, donne into Latin by Sir George Ent, as also his booke de Generatione Animalium, but a little booke in 12mo. against Riolan (I thinke) wherein he makes out his doctrine clearer, was writt by himselfe, and that, as I take it, at Oxford.

His Maj. K. Cha. I. gave him the wardenship of Merton Colledge, as a reward for his service, but the times suffered him not to receive or enjoy any benefit by it.

He was physitian and a great favourite of the Lord High Marshall of England, Tho. Howard, Earl of Arundel and Surrey, with whom he travelled as his physitian in his ambassade to the Emperor ———, at Vienna, Ao. Dni. 163–. Mr. Hollar (who was then one of his excellencie's gentlemen) told me, that in his voyage, he would still be making of excursions into the woods, making observations of strange trees and plants, earths, &c. and sometimes like to be lost. So that my Lord Ambassador would be really angry with him, for there was not only danger of thieves but also of wild beasts.

He was much and often troubled with the gowte, and his way of cure was thus: he would then sitt with his legges bare, if it were frost, on the leads of Cockaine-house, putt them into a payle of water, till he was almost dead with cold, and betake himselfe to his stove, and so 'twas gone.

He was hott headed, and his thoughts working would many times keep him from sleeping: he told me, that then his way was, to rise out of his bed and walke about his chamber in his shirt, till he was pretty coole, i. e. till he began to have a horror, and then returne to his bed, and sleep very comfortably.

I remember he was wont to drinke coffee, which he and his brother Eliab did, before coffee-houses were in fashion in London.

All his profession would allowe him to be an excellent anatomist, but I never heard any that admired his therapeutique-way. I knew several practitioners in this towne (London) that would not have given 3d for one of his bills; and that a man could hardly tell by one of his bills what he did aime at.

He did not care for chymistrey, and was wont to speak against them,* with undervalue.

It is now fitt, and but just, that I should endeavour to undeceive the world in a scandall, that I find strongly runnes of him, wch. I have mett amongst some learned young men: viz. that he made

* Sic Edit.

himselfe himselfe away, to putt himselfe out of his paine, by opium; not but that, had he laboured under great paines, he had been readie enough to have donne it, I doe not deny, that it was not according to his principles upon certain occations to ———, but the manner of his dyeing was really and bonâ fide thus, viz. the morning of his death, about 10 o'clock, he went to speake, and found he had the dead palsey in his tongue, then he sawe what was to become of him, he knew there was then no hopes of his recovery, so presently sends for his young nephews to come up to him, to whom he gives one his watch,* to another another remembrance, &c. made sign to ——— Sambroke, his apothecary, in Black-fryars, to lett him blood in the tongue, which did little or no good, and so he ended his dayes. His practice was not very great towards his latter end, he declined it, unlesse to a speciall friend,—e. g. my Lady Howland, who had a cancer in her breast, which he did cut off and seared, but at last she dyed of it.

He rode on horseback with a foot-cloath to visitt his patients, his man following on foot, as the fashion then was, wch. was very decent, now quite discontinued. The judges rode also with their foot-cloathes to Westminster-hall, wch. ended at the death of Sir Rob. Hyde, Lord Ch. Justice. Anth. E. of Shaft. would have revived [it], but several of the judges being old and ill horsemen would not agree to it. The scandall aforesaid is from Sir Charles Scarborough's sayings that he had, towards his latter end, a preparation of opium, and I know not what, which he kept in his study to take, if occasion should serve, to putt him out of his paine, and which Sir Charles promised to give him: this I believe to be true; but do not at all believe that he really did give it him. The palsey did give him an easie passeport.

---

*An Account of some Experiments on Animal Heat; by* John Davy, M. D. F. R. S.—The recent inquiries of Mr. Brodie have rendered questionable the different prevailing hypotheses relative to animal heat, and have shown that fresh investigation is necessary, before we can expect to arrive at any accurate theory.

In the present uncertain state of our knowledge, three circumstances are particularly deserving of attention, viz. the relative capacities of venous and arterial blood for heat, their comparative temperatures, and the temperatures of different parts of the animal body.

On the first of these subjects we possess only the experiments of Dr. Crawford, which I believe have not yet been repeated, notwithstanding they form the basis of his hypothesis.

On the second, little inquiry has been made, and especially of late years, since the improvement of the thermometer.

---

* 'Twas a minute watch, wth. wch. he made his experiments.

And on the third, the observations that have been collected are very few in number, and, with the exception of those of Messrs. Hunter and Carlisle, are scarcely, perhaps, deserving of confidence.

Such were the inducements that led me to the consideration of each of these subjects apart, and to endeavour to acquire by experiment some more certain knowledge respecting them. The experiments that I have made will be described in the two following sections, and in the last will be offered the few remarks and conclusions which naturally arise, and are fairly deducible from the results.

1. *On the Capacities of venous and arterial Blood for Heat.*

I must premise, that my object has been to endeavour to ascertain the relative capacities of venous and arterial blood for heat, rather than their exact specific caloric. The latter, from many circumstances, is difficult to be accomplished; whilst the former is comparatively easy, and in a theoretical point of view is probably equally useful.

I have employed both the methods commonly used. I shall mention most of the experiments that I have made, without noticing the repetitions of them, and shall begin with those on the times of cooling of equal volumes of venous and arterial blood.

The blood used was from the jugular vein and the carotid artery of a lamb about four months old. It was received in bottles; and, to remove the fibrin, which is a great impediment in experiments of this kind, it was immediately stirred with a wooden rod. In respect to colour, the difference between the venous and arterial blood was not so great as in the sheep's; and this in a great variety of instances I have always observed, the venous being of a less dark hue. The specific gravity of the venous blood, without the fibrin, was found to be 1050, and that of the arterial 1047.

A glass bottle equal in capacity to 2518 grains of water, and weighing 1332 grains, was filled respectively with water and venous and arterial blood of the temperature of the room 62, about four hours after the blood had been drawn, during which time each bottle had been closely corked. A delicate thermometer, by means of a perforated cork, was placed in the middle of the liquid. The bottle was then plunged into water of the temperature 140 Fahrenheit; and, when the mercury had risen to 120, the bottle was quickly wiped and suspended in the middle of the room, and the progress of cooling was noticed every five minutes, till the thermometer had fallen to 80. The following were the general results obtained:

Water cooled from 120 to 80 in 91 minutes
Arterial blood in -- -- -- 89
Venous blood in -- -- -- 88

Considering therefore the capacity of water for heat to be denoted by 1000, neglecting the effect of the glass bottle producing a difference only of about half a minute, and the same in each instance, and dividing the times of cooling by the specific gravity, the relative capacities of venous and arterial blood without fibrin appear to be as ·921 and ·934.

In the following experiments the same kind of blood and the same quantity was used as in the preceding. The mixtures were made in a very thin glass receiver, containing a delicate thermometer. The temperature of the room was 66.

Hot water temperature 121; cold water 61. Mixture of the two 90, after two minutes 89, after three 88, and after eight 87.

Venous blood 121. Water 62·5. Mixture 89; after three minutes 88·5; after seven 87.

Arterial blood 121. Water 63·5. Mixture 89·5; after three minutes 88·5, and after seven 87.

Now, allowing about one degree of the cooling effect to have been produced by the receiver indicated by the admixture of the hot and cold water, calculating the quantity of blood used from the knowledge of its volume and specific gravity, employing the formula given by Professor Robison, which consists in multiplying respectively the weight of the water and the blood by the change of temperature, and dividing the first product by the second, the quotient or specific caloric for venous blood appears to be as ·812, and for arterial as ·814, results very similar to those I have obtained with the blood of the sheep.

In the remaining experiments, blood with the fibrin present was employed, and with this exception they were perfectly similar to those already described.

The blood used to ascertain its time of cooling was obtained from a sheep; and one day the vein was opened, and on the next the artery. The capacity of the bottle employed exceeded that of the first by one ounce measure of water; but it was equally thin. The air of the room was of temperature 69.

Water cooled from 120 to 80 in 118 minutes
Venous blood in -- -- -- 112
Arterial blood in -- -- -- 113

And hence, as the latter was of specific gravity 1049, its capacity for heat seems to be as ·913; and as the former was of specific gravity 1051, its capacity appears to be ·903.

In the following experiment, equal volumes of fluid blood and of water were used; which was easily accomplished by means of a thin bottle with a large mouth, to which a cork was adapted, with a perforation more than sufficiently large to admit the bulb of a very delicate thermometer, and of course to allow, when the bottle was filled to the brim, the excess to flow out on the introduction of the cork, which was always similarly placed. To retard the process of cooling, the bottle was closely surrounded by a thick layer of what is commonly called cotton-wool. Its capacity was equal to five ounce measures of water, or 2400 grains. It was first filled with cold water, which, when its temperature had been ascertained, was thrown into the receiver

 before before used; it was next filled with hot water of temperature about 110, so that the heat of the glass might be nearly the same as that of the blood: and lastly, when the vein or artery had been opened, the bottle was immediately emptied and filled with blood, the temperature of which was ascertained by the thermometer in less than a quarter of a minute. The mixture now was instantly made, and by the same thermometer the highest temperature after mixture was discovered.

The four following trials were made on the blood of two lambs, both about five months old. The temperature of the air was 60.

Cold water 57·5. Venous blood 100. Mixture 80; after one minute 78·5.

Water 58. Arterial blood 103. Mixture 80; after one minute 79.

Water 58. Venous blood 101·5. Mixture 79; after one minute 78·25.

Water 58. Arterial blood 106·5. Mixture 81; after one minute 80.

The rate of cooling was not noticed after the first minute had elapsed, as the blood then generally began to coagulate. The specific gravity was not only ascertained in the two last trials; that of the venous blood was found to be 1050, and that of the arterial 1049; and hence allowing, as before, one degree of the cooling effect to be produced by the receiver, the capacity of venous blood for heat appears to be ·852, and that of arterial blood ·839.

It is evident that these trials admit of less accuracy than the preceding; and much more confidence, it appears to me, is due to the third series of experiments; so that, if required, I should be inclined to give the numbers thence deduced, as the greatest approximation to the truth.

2. *On the comparative Temperatures of venous and arterial Blood, and of differeut Parts of the animal Body.*—To endeavour to ascertain the comparative temperature of venous and arterial blood, I have a considerable number of experiments; some of which on lambs, sheep, and oxen, it will be sufficient for me in this place to describe. In each instance, a long incision was made through the integuments; the jugular vein was laid bare, and the exact seat of the carotid artery found. The vein was then opened, and a small delicate thermometer introduced, and thrust about an inch up the vessel beyond the wounded part; and as the bulb of the instrument was small, the flow of blood was not stopped. When the mercury was stationary, its height was marked. The carotid artery next was divided, and the thermometer was immersed in the current of blood, and left there till it ceased to rise.

The following are the results of five experiments made on lambs, all of which were about three months old. The thermometer in the shade stood at 65.

1. Venous

| | | | |
|---|---|---|---|
| 1. Venous blood | 102·5 | Arterial blood | 104 |
| 2. ——— | 104 | ——— | 105 |
| 3. ——— | 104 | ——— | 105 |
| 4. ——— | 103.5 | ——— | 105 |
| 5. ——— | 104 | ——— | 105 |

The following results were obtained from three experiments on sheep, whose exact age I could not ascertain. The thermometer in the shade was 60.

| | | | |
|---|---|---|---|
| 1. Venous blood | 103·5 | Arterial blood | 104·5 |
| 2. ——— | 102·5 | ——— | 104 |
| 3. ——— | 103 | ——— | 104 |

The experiments on oxen were only two in number. The temperature of the air was 64.

| | | | |
|---|---|---|---|
| 1. Venous blood | 100 | Arterial blood | 101·5 |
| 2. ——— | 100 | ——— | 101 |

In both instances the oxen were knocked down before the vessels were opened; and as respiration had ceased in consequence of the injury of the brain and spinal cord, no difference of colour, of course, was preceptible between the blood from the jugular vein and that from the carotid artery.

These results, so different from what might have been expected from the observations of Messrs. Coleman and Cooper on the temperature of the two sides of the heart, led me to repeat their experiments. The experiments in which I place most confidence were made on lambs about four months old, and to these I shall confine myself at present. In each instance the animal was killed by the division of the great vessels of the neck; an opening was made immediately into the thorax, and a very delicate thermometer was introduced into the ventricles of the heart by means of a small incision. The operation occupied so short a space of time, that in three instances the right auricle had not ceased contracting.

| | | | |
|---|---|---|---|
| 1. Venous blood | 104 | Left | 106 |
| Arterial | 105·5 | 3. Rectum | 105 |
| Rectum | 104 | Right ventricle | 105·5 |
| Right ventricle | 105·5 | Left | 106·5 |
| Left | 106 | 4. Rectum | 105 |
| 2. Rectum | 105 | Right ventricle | 106 |
| Right ventricle | 105 | Left | 107. |

I cannot well explain the difference which exists between the results of the preceding experiments, and those of Messrs. Coleman and Cooper, which are directly opposite. Can the mode in which the animals they experimented on where put to death, be the cause of the want of agreement? In death by asphyxia, there is generally an accumulation of blood in the right ventricle; and in many instances I have observed, when the right ventricle has been distended with blood, little difference of temperature between the two sides of the heart.

To describe all the experiments that I have made to ascertain the temperature of the different parts of the animal body, would extend this paper to an unusual length, and there is the less occasion casion for a long description, as the comparative results were very similar. It will be sufficient therefore here to notice the observations made on the human body, and on that of a lamb.

That the thermometer might be equally applied to all parts of the surface, its bulb, in form nearly cylindrical, was fixed to a small piece of cork, hollowed and lined with fine wool, and thus half its superficies was applied in each instance. The observations were made on the naked body at seven A. M. immediately after quitting bed. The temperature of the air of the room was 70. The following were the results obtained:

| | |
|---|---|
| At the central part of the sole of the foot | 90 |
| Between the malleolus internus and the insertion of the tendo Achillis, where the artery is felt | 93 |
| Over the middle of the tibia | 91·5 |
| On the middle of the calf | 93 |
| Over the popliteal artery at the bend of the knee | 95 |
| Over the femoral artery in the middle of the thigh | 94 |
| Over the middle of the rectus muscle | 91 |
| Over the great vessels in the groin | 96·5 |
| About a quarter of an inch below the umbilicus | 98 |
| Over the sixth rib on the left side where the heart is felt pulsating | 94 |
| Over the same place in the right | 93 |
| Under the axilla, the whole surface of the bulb being applied | 98 |

About an hour had now elapsed from the commencement of the experiment. The thermometer again applied to the sole of the foot rose no higher than 85, five degrees less than at first. A disagreeable sensation of cold was experienced, and particularly in those parts not supplied with large vessels, and out of the course of the great arteries. The body remained unpleasantly chilly till breakfast had been taken, and then a slight degree of pyrexia was perceived; the heat of surface being increased, the pulse quickened, and the mouth slightly parched. After breakfast, the thermometer was applied to both hypochondriac regions, and the left was found one degree higher than the right.

To ascertain the temperature of different parts of the surface beneath the integuments, the bulb of a thermometer was introduced through small incisions about half an inch between the skin and subjacent parts of a lamb just dead. The heat of the rectum was first ascertained, as a means of marking the rate of cooling, and the different parts were then tried in the following order:

| | |
|---|---|
| Venous blood in the jugular vein | 105·5 |
| Arterial blood from the carotid artery | 107 |
| Rectum | 105.5 |
| Over the metatarsal bone | 97 |
| Over the tarsal bone | 90 |
| Over the knee joint | 102 |

About

| | |
|---|---|
| About the head of the thigh | 103 |
| At the groin | 104 |

Nearly a quarter of an hour had been occupied in making these observations, and the temperature of the rectum was now found to be 105. The three great cavities were next opened in the order enumerated.

| | |
|---|---|
| Near the lower part of the liver | 106 |
| The substance of the liver | 106.5 |
| The substance of the lung | 106·5 |
| The left ventricle | 107 |
| The right ventricle | 106 |
| The central substance of the brain | 104 |
| Rectum | 104·5 |

Surprised at the temperature of the brain being lower than that of the rectum, I was led to repeat the experiment. It may be proper to notice a few of the results, as it is a curious circumstance which they confirm. The four experiments I shall mention were made on lambs. As soon as the animal was dead, the cranium was perforated, and a delicate thermometer introduced into the central part of the brain.

1. Brain 104 Rectum 104.75
2. Brain 104·75 Rectum 105·5
3. Brain 105·5 Rectum 106·5
4. Posterior part of the brain 105·5; anterior 103. Rectum 106·5.

The temperature of the air at the time was 68. Different parts of the brain were found to vary considerably in temperature; the anterior, as already noticed, being lower than the posterior, and the superficial than the deep-seated parts.

### 3. *Remarks and Conclusions.*

That there is no material difference between venous and arterial blood in respect to specific caloric, excepting what arises from difference of specific gravity; that the temperature of arterial blood is higher than that of venous; and the temperature of the left side of the heart, than that of the right; and lastly, that the temperature of parts diminishes as the distance of the parts from the heart increases—are the general results of the preceding experiments.

Admitting the accuracy of these experiments, and I think that they will be found correct when repeated, what are their consequences in a theoretical point of view?

They are evidently in direct opposition to Dr. Crawford's hypothesis; the essence of which is, that the capacity of arterial blood for heat is greater than that of venous, that there is no difference of temperature between the two ventricles of the heart, and in fact that the heat of all parts is nearly the same.

They are more agreeable to, and indeed they even support, the hypothesis of Dr. Black, that animal heat is produced in the lungs, and distributed over the whole system by means of the arterial blood.

Neither

Neither are they inconsistent with that hypothesis which considers the production of animal heat as dependant on the energy of the nervous system, and arising from all the vital actions constantly occurring.

Besides the results of the preceding experiments, many arguments may be advanced in opposition to Dr. Crawford's hypothesis.

As we never perceive a difference of capacity in bodies without a difference of form or composition; and as very slight differences of the former result only from great changes of the latter: it might be expected *a priori*, as no difference, excepting that of colour, has been detected between venous and arterial blood, that their specific caloric would be very similar. From analogy also, it might have been expected, that the capacity of arterial blood for heat would be much less than that of water, as water appears to exceed almost every other fluid, and as the capacity appears to diminish as the inflammability of compounds increases. But the strongest arguments against this hypothesis are to be derived from the recent experiments of Mr. Brodie, and those of MM. Delaroche and Berard.

Dr. Black's hypothesis appears to me far more satisfactory than Dr. Crawford's, and capable of explaining a much greater number of phænomena; but there are objections even to this hypothesis, which must be removed before it can with propriety be received.

The last hypothesis, which I mentioned, that which refers animal heat to vital action, has many facts in its support, and especially the results of Mr. Brodie's curious and interesting experiments; and the results of my inquiry, as I have already observed, are not incompatible with it. It may be said, that the viscera of the thorax and abdomen are of highest temperature, because these parts are, as it were, the elaboratories of life; and that the heat of the arterial blood, and of the parts best supplied with this fluid, is greatest, because they lie deepest and abound most in the principle of life or vital action. This explanation was suggested to me by my brother Sir H. Davy. There are some facts which I have observed agreeable to it, but not more so than to the hypothesis of Dr. Black. I have found the stomach of the ox, the pyloric compartment, of a higher temperature than the left ventricle itself: thus, when the latter immediately after death was 103, the former full of food was 104·5. I have also found the temperature of young animals, in whom all the vital actions are most energetic, higher than that of animals arrived at maturity. I may mention here, in illustration of this statement, a few observations made on infants, as I am not acquainted with any yet published. In one instance I found the heat under the axilla of a child just born 98·5; after twelve hours 99, and after three days, the same; during the whole of which time it appeared in perfect health. On five other children of the same age, similar observations were made. In two instances of of weak infants, the temperature, one hour after birth, was found not to exceed 96, which is two degrees below the standard heat of man in a state of health; but their respiration was still languid, and the next day the heat of the axilla had risen in one to 98·5, and in the other to 99.

To conclude: As, in each hypothesis examined, difficulties are found to exist from facts or the results of experiments of an unbending nature, we must at present either suspend theory altogether and search for *experimenta crucis*, or adopt that hypothesis which is conformable to the greater number of facts. The first measure is certainly most philosophical; but to the latter we are naturally most inclined; and if I were questioned which view is preferable, I should make no hesitation in selecting Dr. Black's, which to me appears both most simple and most satisfactory.—*Phil. Trans.*

---

### *Some Experiments on the Combustion of the Diamond and other carbonaceous Substances.* *By* Sir Humphry Davy, LL.D. F.R.S. V.P. R.I.

During a stay that I made at Florence in the end of March and beginning of April, I made several experiments on the combustion of the Diamond, and of plumbago, by means of the great lens in the Cabinet of Natural History; the same instrument as that employed in the first trials on the action of the solar heat on the diamond, instituted by Cosmo III. Grand Duke of Tuscany; and I have since made a series of researches on the combustion of different kinds of charcoal at Rome, in the laboratory of the Academia Lyncei. In the first series I was honoured by the assistance of the Count Bardi, the Director, and Signior Gazzari, the Professor of Chemistry at the Florentine Museum: and in the last by that of Sig. Morrichini and Barlocci, Professors of the College Sapienza at Rome.

In the very first trials on the combustion of the diamond, I ascertained a circumstance that I believe has not been noticed before; namely, that the diamond, when strongly ignited by the lens in a thin capsule of platinum perforated with many orifices, so as to admit a free circulation of air, continues to burn in oxygen gas after being withdrawn from the focus. The light it affords is steady, and of so brilliant a red, as to be visible in the brightest sunshine; and the heat produced is so great, that in one experiment, in which three fragments of diamonds weighing 1·84 grain only were burnt, a fine wire of platinum used for attaching them to the tray was fused, and that some time after the diamonds were removed out of the focus.

The knowledge of this circumstance enabled me to adopt a very simple apparatus and mode of operation in my researches, and to complete, in a few minutes, experiments which have been supposed to require the presence of a bright sunshine for many hours.

My apparatus consisted of clear glass globes of the capacity of from fourteen to forty cubical inches, having single apertures to to which stop-cocks were attached: a small hollow cylinder of platinum, which I use in experiments with the blow-pipe, was attached to one end of the stop-cock, and was mounted with a little perforated capsule of platinum for containing the diamond. When the experiment was to be made, the globe containing the capsule and the substance to be burned was exhausted by an excellent air-pump, and pure oxygen gas, made from hyperoxymuriate of potassa, admitted. The globe before and after the experiment was brought to the same temperature as the water over which the oxygen gas had remained. And, as during the short time required for the combustion there was no sensible change either in the thermometer or barometer, no corrections for pressure or temperature were rendered necessary; the change of volume in the gas after the combustion, was estimated by means of a fine tube connected with a stop-cock, adapted by a proper screw to the stop-cock of the globe, and the absorption was judged of by the quantity of mercury that entered the tube, which afforded a measure so exact that no alteration, however minute, could be overlooked. As the elastic force of the vapour of water is the same at the same temperature, it was evident, that if any water formed in these experiments, it would be deposited as dew or mist in the globe; and I am convinced by direct trials, that a quantity of moisture not capable of being weighed by a balance sensible to the $\frac{1}{100}$ of a grain, is rendered evident by deposition on a polished glass surface.*

The diamonds were always heated to redness before they were introduced into the capsule.

During the combustion of the diamond, the glass globe was kept cool by the application of water to that part of it immediately above the capsule, and where the heat was the greatest.

In the first experiment, three diamonds, weighing together 1·63 grain, were entirely consumed, in a quantity of oxygen gas, more than three times as much as was necessary to convert them into carbonic acid. In this case, after the combustion had once commenced, it continued without a fresh application of the lens till there remained only a very thin piece of the largest diamond in contact with the capsule, and this by being brought into the focus rapidly disappeared. On restoring the globe to its original temperature, there was a very evident deposition of moisture; but on arranging the apparatus, so as to ascertain the change of volume of the gas, there entered only twenty-one grains of mercury. In this experiment, the cylinder of platinum

* A piece of paper weighing a grain was introduced into a tube of about the capacity of four cubical inches, the exterior of which was gently heated by a candle; immediately a slight dew was perceptible in the interior of the upper part of the tube; the paper taken out and weighed immediately in the balance above referred to, had not suffered any appreciable diminution.

had had been fastened into the stop-cock by means of a small perforated cork: it seemed probable, when the small diminution of gas was considered, that the appearance of moisture might be owing to the production of vapour from this cork during the combustion, and the second experiment demonstrated that this was the case.

In this second experiment 1·84 grain of small diamonds were employed, and a glass globe of the capacity of 14·9 cubical inches. Soon after the capsule was placed in the focus in bright sunshine, the diamonds burnt with great brilliancy, and continued to burn till they had considerably diminished in bulk; but their splendour of combustion gradually became less, and before they had apparently lost half of their volume the process ceased. By placing them a second time in the focus, after agitating the globe so as to change their places, the combustion was again produced; but the light was much less vivid than before, and the combustion continued for a much shorter time. They were exposed to the concentrated rays a third and a fourth time; but after the fourth time they seemed incapable of burning, and though kept for some minutes in the focus, appeared to undergo no further diminution: two fragments remained, which, as it was afterwards found, weighed ·52 of a grain; the barometer during the experiment was at 29·9 inches, the thermometer at 56° Fahrenheit. When the original temperature of the globe was restored, there was not the slightest appearance of vapour or humidity; the interior was as clear as before the experiment, and there was no solid matter of any kind separated in the tray. The fragments of diamond which remained were not black, but had lost their lustre like glass that has been acted on by fluoric acid, nor at any period of the process was any carbonaceous appearance perceived upon them. When the communication was made by the stop-cock between the interior of the globe and a surface of mercury, a minute quantity entered equal to 1·5 grain only.

A portion of the gas in the globe was transferred into a tube in the mercurial apparatus, and the oxygen it contained absorbed by the combustion of phosphorus; 3·5 parts of gas heated in this way left a residuum of 2·5 parts. A portion of the gas was agitated with lime-water, when seven parts out of ten were absorbed. I exposed the gas which remained after the combustion of phosphorus to several tests; it had not only the obvious characters of carbonic acid, but exhibited exactly the same chemical phænomena. Potassium strongly heated in it in a small glass tube over mercury, burnt with a dull red light, and formed an alkaline product of the same intense black colour as that produced by its combustion in the carbonic acid procured by the dissolution of marble: distilled water absorbed rather less than its own volume of the gas, and became subacid, sparkled by agitation, gained the taste and smell of a solution of carbonic acid in water, precipitated in the same manner limewater, and when in excess redissolved the precipitate. To ascertain if this precipitate was exactly the same in composition as pure carbonate of lime, I made a sufficient quantity of it by pouring lime-water into the recipient containing the results of the first experiment; and after collecting and drying it at the temperature of 212° Fahrenheit, I introduced a quantity of it contained in some foil of platinum through mercury into a glass tube filled with mercury, and I heated in the same manner an equal quantity of finely powdered Carrara marble, and admitted to them equal quantities of solution of muriatic acid. In this trial, there was rather more elastic fluid disengaged from the Carrara marble than from the carbonate of lime from the diamond; but on examining the foil of platinum after the experiment, I found that a little of the carbonate had not been acted upon: I tried two similar experiments, substituting bibulous paper instead of the metallic foils for infolding the carbonates; the results were such as to show that both substances afforded the same quantities of elastic fluid.

I heated some of the carbonate from the diamond in a tube which contained potassium, and passed the potassium through it in vapour: there was ignition, and a substance of a dense black colour was formed; this substance was acted on by dilute muriatic acid, when it left a fine black powder which burnt like lamp black, and when thrown into fused nitre scintillated and disappeared in the same manner as powdered charcoal.

The gas that remained in the second experiment, after the absorption of the carbonic acid gas, vividly supported combustion, and diminished with nitrous gas; but as the degree of purity of the oxygen gas with which the globe was filled had not been determined before the experiment, it was impossible to ascertain with precision, that no elastic matter had been emitted during the process. To determine this point, I made a third experiment. A thin diamond weighing ·93 of a grain was introduced into the platinum capsule, which was placed in a globe filled with water and inverted in water; some oxygen gas, the last portion from the decomposition of hyper-oxymuriate of potassa, was thrown into the globe, so as to displace the water below the level of the capsule. The focus of the great lens was thrown upon the capsule, which with the diamond was instantly rendered dry by it, and the diamond soon entered into combustion and burnt as usual. After the process was finished, the carbonic acid was absorbed by lime-water, and the remaining gas, which equalled about one-third of the quantity of oxygen originally used, was compared analytically in several experiments with a portion of the same oxygen as that introduced into the globe, two measures of nitrous gas being added to a measure of each of the gases; the diminution was less by from $\frac{1}{100}$ to $\frac{2}{100}$ parts in the cases in which the gas that had been exposed to the action of the diamond was used; but this minute difference is what might have been expected, and which indeed could not fail to exist, when it is considered that, during the absorption sorption of carbonic acid gas by water and lime water, a small quantity of common air is always expelled from the water.

In this last experiment a small fragment of diamond remained unconsumed, which was similar in appearance to that mentioned in the second experiment, and its colour, which was originally yellow, was rendered rather darker. In no one of the three experiments was there any distinct appearance of carbonization, when the process was stopped in consequence of the impurity of the gas; though the diamonds were of various colours and different lustres.

A piece of plumbago from Borrowdale in Cumberland, weighing two grains, was exposed in the focus in the same manner as the diamond in the first and second experiments, having been previously heated red; the quantity of oxygen gas employed was 8·5 cubical inches: more than half the plumbago remained unconsumed, and during the combustion some brown ashes were produced. The phænomena in this experiment were very different from those observed in the experiments on the diamond, the gas became clouded during the process, and there was a considerable deposition of dew on the interior of the globe. When the original temperature of the globe was restored, and the stop-cock opened, 96·6 grains of mercury entered, and drops of moisture even were observed condensed on the sides of the vessel.

In the second series of experiments, charcoal formed by the action of sulphuric acid on oil of turpentine, and some produced during the formation of sulphuric ether, from which nitric acid had been distilled, and which afterwards had been strongly ignited, and charcoal of oak which had undergone the same process, were used.

Three grains of the charcoal from turpentine were employed, 2·5 of that from alcohol, and five grains of the charcoal of the oak: in all these instances of combustion the gas became clouded during the combustion, and, when the original temperature of the globe was restored, moisture was found condensed in the interior; much the largest quantity in the experiment on the charcoal of oak, and the least in that on the charcoal procured from oil of turpentine. The charcoal from oak left a residuum of white ashes, which was principally carbonate of lime; that from oil of turpentine produced no residuum; that from alcohol, which was formed in a common process of the manufacture of ether, left a minute quantity of ashes, probably owing to the impurity of the sulphuric acid employed.

The quantity of mercury which entered the apparatus indicating the change of volume of the gas, was in the experiment on the charcoal of oil of turpentine .......................... 107·5 grains

In that on the charcoal of alcohol .............. 194·5

In that on the charcoal of oak.................... 513·3

From the results of these different experiments, it appears evident, that the diamond affords no other substance by its combustion than pure carbonic acid gas; and that the process is merely a solution of diamond in oxygen, without any change in the volume of the gas; for the slight absorption in the second experiment is scarcely more than a compensation for the volume occupied by the diamonds consumed.

It is likewise evident that in the combustion of the different kinds of charcoal, water is produced; and from the diminution of the volume of the gas, there is every reason to believe, that the water is formed by the combustion of hydrogen existing in the charcoal; and experiments which I have referred to, or detailed in my third Bakerian Lecture, prove the presence of hydrogen in common charcoal; and as the charcoal from the oil of turpentine left no residuum, no other cause but the presence of hydrogen can be assigned for the diminution occasioned in the volume of the gas during its combustion.

M. Guyton de Morveau* has noticed the production of water during the combustion of plumbago from Keswick; and from these experiments it is most probable that it is formed in the process of combustion, for it is unlikely that water should remain in union with plumbago at a red heat; and in the various experiments that I have made on the ignition of plumbago by Voltaic electricity, I have never perceived the separation of any moisture, or the production of any gas; so that it seems most likely that it contains intimately combined hydrogen. It cannot be supposed that water exists in it in union with oxide of iron, for in this case there would be no obvious cause for the diminution of the volume of the gas; and all analogy is in favour of the conclusion that the iron is in a metallic state.

The general tenor of the results of these experiments is opposed to the opinion, that common carbonaceous substances differ from the diamond by containing oxygen; for in this case they ought to increase and not diminish the volume of oxygen; nor, on the other hand, is it favourable to the supposition that the diamond contains oxygen, for the difference in the quantity of carbonic acid produced in the different experiments, is no more than may be reasonably ascribed to the generation of water, in the combustion of the common carbonaceous substances; and the results of the experiment., to which I have referred in the beginning of this paper on the action of potassium on the diamond, may be easily accounted for from other circumstances.†

That charcoal is more inflammable than the diamond, may be explained from the looseness of its texture, and from the hydro-

---

* Annales de Chimie, tome lxxxiv. p. 241.

† See Bakerian Lecture for 1808. Potassium decomposes the silica in glass by being heated in contact with it; and in the case in which equal quantities of potassium were long heated in glass tubes, the one in contact with diamonds, the other alone, that in contact with the diamonds must have acted upon a much greater surface of glass.

gen it contains; but the diamond appears to burn in oxygen with as much facility as plumbago, so that at least one distinction supposed to exist between the diamond and common carbonaceous substances is done away by these researches.

A fact which I formerly noticed, the blackening of diamond by the long continued action of heated potassium, induced me to suspect in the beginning of these inquiries, that common charcoal might owe its colour, opacity, and conducting power, to the circumstance of its containing minute portions of the metals of the alkalies, or earths, and plumbago to the iron it contains; but when I found that charcoal made from oil of turpentine, which left no residuum in burning, aud charcoal precipitated from carburetted hydrogen gas by chlorine, had the same properties, it was necessary to renounce this opinion.

The only chemical difference perceptible between diamond and the purest charcoal, is, that the last contains a minute portion of hydrogen; but can a quantity of an element, less in some cases than $\frac{1}{50000}$ part of the weight of the substance, occasion so great a difference in physical and chemical characters? this is possible, yet it is contrary to analogy; and I am more inclined to adopt the opinion of Mr. Tennant, that the difference depends upon crystallization. Transparent solid bodies are in general non-conductors of electricity, and it is probable that the same corpuscular arrangements which give to matter the power of transmitting and polarizing light, are likewise connected with its relations to electricity; and water, the hydrates of the alkalies, and a number of other bodies which are conductors of electricity when fluid, become non-conductors in their crystallized form.

The power possessed by certain carbonaceous substances of absorbing gases, and separating colouring matters from fluids, is probably mechanical and dependent upon their porous nature; for it belongs in the highest degree to vegetable and animal charcoal, and it does not exist in plumbago, coke, or anthracolite.

The same conclusions respecting the composition of carbonic acid may be drawn from these experiments, as from those of Messrs. Allen and Pepys, and Theodore de Saussure. If the calculations be founded upon the difference of the weights of oxygen and carbonic acid gases, which appears the most exact method, carbonic acid gas will contain, according to the estimate of the mean specific of the gravities of the two gases given by M. Theodore de Saussure,* thirty parts of oxygen, or two definite proportions, to 11·3 parts of carbon, or one definite proportion.

Supposing the diminution of the oxygen produced in the experiments on the common carbonaceous substances entirely occasioned by the formation of water, it is easy to calculate the proportions of hydrogen in them; but in the case of plumbago there is probably

* Annales de Chimie, tome lxxi. pag. 261. This estimation is the same as that I have given. Elements of Chem. Phil. pag. 305.

a diminution a diminution of oxygen, from the oxidation of the iron; and it is not certain that the ashes afforded by the charcoal from vegetable substances exist in it in the state of earths and alkalies: and as the quantity of hydrogen varies according to the degree of heat to which charcoal has been exposed, it is almost useless to attempt to assign its proportion in any particular case, especially when the largest portion is so extremely minute.

The nature of the chemical difference between the diamond and other carbonaceous substances may be demonstrated by another process, namely, igniting them in chlorine; when common well burnt charcoal, or plumbago from Cumberland, is intensely ignited in chlorine, white fumes are immediately perceived in consequence of the production of muriatic acid gas by the hydrogen, which acid precipitates the aqueous vapour in the chlorine; but the diamond occasions no such effect. A small diamond, weighing ·45 of a grain, was kept in a state of intense ignition by the great lens of the Florentine Museum for more than half an hour; but the gas suffered no change, and the diamond had undergone no diminution of weight, and was not altered in appearance.

Charcoal, after being intensely ignited in chlorine, is not altered in its conducting power or colour; and this circumstance is in favour of the opinion, that the minute quantity of hydrogen is not the cause of the great difference between the physical properties of the diamond and charcoal.—*Phil. Trans.* 1814. *Part II.*

